# A tabletability change classification system in supporting the tablet formulation design via the roll compaction and dry granulation process

**DOI:** 10.1016/j.ijpx.2023.100204

**Published:** 2023-07-28

**Authors:** Junhui Su, Kunfeng Zhang, Feiyu Qi, Junjie Cao, Yuhua Miao, Zhiqiang Zhang, Yanjiang Qiao, Bing Xu

**Affiliations:** a Department of Chinese Medicine Informatics, Beijing University of Chinese Medicine, Beijing 100029, PR China; b Beijing Key Laboratory of Chinese Medicine Manufacturing Process Control and Quality Evaluation, Beijing 100029, PR China; c The International Department, No. 8 Middle School of Beijing, Beijing 100045, PR China; d Beijing Tcmages Pharmceutical Co. LTD, Beijing 101301, PR China

**Keywords:** Tabletability change classification system (TCCS), Material library, Roll compaction and dry granulation (RCDG), Formulation design, Risk decision tree

## Abstract

In this paper, the material library approach was used to uncover the pattern of tabletability change and related risk for tablet formulation design under the roll compaction and dry granulation (RCDG) process. 31 materials were fully characterized using 18 physical parameters and 9 compression behavior classification system (CBCS) parameters. Then, each material was dry granulated and sieved into small granules (125–250 μm) and large granules (630–850 μm), respectively. The compression behavior of granules was characterized by the CBCS descriptors, and were compared with that of ungranulated powders. The relative change of tabletability (*CoT*_r_) index was used to establish the tabletability change classification system (TCCS), and all materials were classified into three types, i.e. loss of tabletability (LoT, Type I), unchanged tabletability (Type II) and increase of tabletability (Type III). Results showed that approximately 65% of materials presented LoT, and as the granules size increased, 84% of the materials exhibited LoT. A risk decision tree was innovatively proposed by joint application of the CBCS tabletability categories and the TCCS tabletability change types. It was found that the LoT posed little risk to the tensile strength of the final tablet, when Category 1 or 2A materials, or Category 2B materials with Type II or Type III change of tabletability were used. Formulation risk happened to Category 2C or 3 materials, or Category 2B materials with Type I change of tabletability, particularly when high proportions of these materials were involved in tablet formulation. In addition, the risk assessment results were verified in the material property design space developed from a latent variable model in prediction of tablet tensile strength. Overall, results suggested that a combinational use of CBCS and TCCS could aid the decision making in selecting materials for tablet formulation design via RCDG.

## Highlights

• A material library was used to uncover the pattern of tabletability change in RCDG.
• The tabletability change classification system (TCCS) under RCDG was built.
• Joint use of CBCS and TCCS enabled risk decision of material's final tabletability.
• The material tabletability design spaces for DC and RCDG were subdivided.

## Graphical abstract

**Figure:**
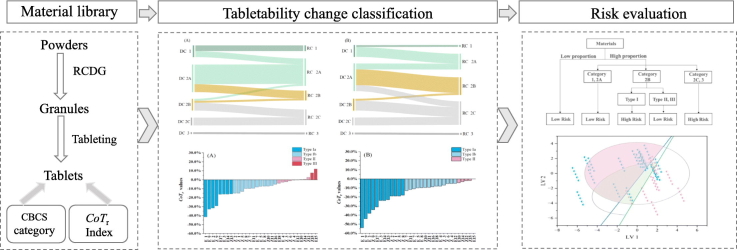

## 1 Introduction

Granulation is important in manufacturing of pharmaceutical oral solid dosage (OSD) forms and possesses the advantages of improving pharmaceutical materials' bulk density and flow properties as well as preventing segregation of active pharmaceutical ingredients (APIs) and dust formation (Parikh, 2021). Among various granulation techniques, roll compaction and dry granulation (RCDG) is widely used in the pharmaceutical industry and consists of a compaction step and a milling step. The main advantages of RCDG are the absence of water or other solvents and cost-intensive drying steps, making this technique feasible for moisture- and/or heat-sensitive materials (Kleinebudde, 2004). Moreover, RCDG is an inherently continuous process which is benefit to pharmaceutical continuous manufacturing (Vervaet and Remon, 2005). Besides a large amount of fines, the main drawback of RCDG is the decrease in tensile strength (*TS*) of tablets produced from dry granules compared to tablets compressed directly from the equivalent powder mixture, which is generally termed as loss of tabletability (LoT) or loss of reworkability (Herting and Kleinebudde, 2008; Malkowska and Khan, 1983; Sun and Kleinebudde, 2016).

Various hypotheses had been proposed to interpret the mechanism of LoT, including the work hardening (Malkowska and Khan, 1983), the granules hardening (Patel et al., 2011) and the particle size enlargement (Sun and Himmelspach, 2006). Malkowska and Khan (1983) first explained the tabletability reduction phenomenon with the work hardening theory and described work hardening as the increased resistance to recompression, because of the entanglement of dislocations on the particle level. However, there were difficulties in demonstrating the presence of lattice dislocations in dry granulation. Instead, Patel et al. proposed the hypothesis of granule hardening, which was mainly related with the reduction of granule porosity (Patel et al., 2011). Sun et al. ascribed the LoT phenomenon of plastic materials to granule size enlargement which led to the reduction in surface area available for bonding in tablet (Sun, 2008; Sun and Himmelspach, 2006). Herting and Kleinebudde (2008) proposed that the LoT was a combination of granules hardening and particle size enlargement. On the basis of previous research, Sun stated that the tensile strength of the tablet was determined by the interaction of the bonding area and bonding strength (BABS) within the tablet, and proposed the use of the BABS principle to understand of the LoT in dry granulation (Sun, 2011). By preparing microcrystalline cellulose (MCC) particles with different porosity structures and studying the loss of their tabletability, Nordstrom and Alderborn (2015) found that the particles with more than a certain porosity almost completely collapsed into primary particles in the subsequent compression process, thus leaving the material tabletability unchanged, and thus proposed the concept of critical porosity. This was further extended by Tofiq et al. that the microstructure of granules resulted from the primary-to-secondary size enlargement process dictated the tableting performance of dry granulated materials (Tofiq et al., 2022a, Tofiq et al., 2022b).

Generally, no single mechanism could explain all observations. Different material properties and process parameters might lead to different deformation behaviors of materials after dry granulation, affecting the dominant mechanism of the LoT after RCDG. In practice, understanding the re-compression properties of different materials is beneficial to designing a balanced and robust formulation. Janssen et al. compared the re-compactibility of 8 materials including 3 types of anhydrous lactose, 3 types of lactose monohydrate and 2 types of MCC, and found that tablet tensile strength of lactose only decreased with 7–29%, while tablet tensile strengths of MCC were decreased by 90% at relatively high specific compaction force of 16 kN/cm (Janssen et al., 2022). Palugan et al. evaluated the compaction ability of 4 grades of mannitol, and it was found that the powder and granular mannitol showed lesser compaction ability than the spray dried mannitol, but the spray dried mannitol were more sensitive in losing tabletability after dry granulation (Palugan et al., 2022). Grote and Kleinebudde studied the influence of 4 kinds of alpha-lactose monohydrate on tablet properties after RCDG (Grote and Kleinebudde, 2019). The results showed that RCDG of lactose monohydrate with *D*_50_ of 131 μm and milled lactose monohydrate with *D*_50_ of 33 μm increased and decreased the tabletability, respectively. Omar et al. revealed that the amorphous part in the lactose powder would crystallize at high relative humidity (RH) value (i.e. 80%), which resulted in the loss of powder compressibility (Omar et al., 2016). The authors suggested that the optimum RH conditions for lactose were in the range of 20–40%. Some excipients with high porosity or small primary particles, such as anhydrous lactose with aggregates of microcrystals (Janssen et al., 2022), agglomerated lactose monohydrate (Grote and Kleinebudde, 2019) and functional calcium phosphate dibasic (DCP) agglomerate (Grote and Kleinebudde, 2018), were sensitive to specific compaction force applied and were suitable for RCDG and further tableting in most cases. Heiman et al. (Heiman et al., 2015) investigated the suitability of roller compaction for high drug loading formulations containing two active pharmaceutical ingredients, and it was shown that the loss in compactibility for the brittle API paracetamol was more pronounced than the plastic API ibuprofen. The possible reasons might be that the granulation of ibuprofen did not generate large particles and the resulted ibuprofen granules were more porous compared to the paracetamol granules.

Recently, the material library or material database approach has been brought forward to speed up the development of formulations and processes for new drug products. This approach mainly involves three steps. The first step is to build a material property database including maximal variability of the underlying raw material dataset. For instance, Basu et al. firstly developed a publicly accessible excipients database named the NIPTE-FDA excipients knowledge base, which provided the formulation scientists with comprehensive properties of >70 commonly used excipients (Basu et al., 2011). Suñé Negre established a material database consisting of 51 directly compressible excipients, and each material was characterized by 12 parameters from the SeDeM expert system (Suñé Negre et al., 2014). Van Snick et al. characterized 55 pharmaceutical powders including 18 APIs and 38 excipients that were commonly used in direct compression (DC) and wet granulation (WG) processes, and each material was described by over 100 material descriptors (Van Snick et al., 2018). In the next step, quantitative correlation models could be constructed by linking physical properties of raw materials to unit operations and final product performance. The materials used in such models could be all materials in the material library, or could be a representative material subset selected by the material sparing approaches (Dhondt et al., 2022b; Wang et al., 2019). Currently, the process models on the basis of the material library approach were mainly implemented on single unit operations, such as the direct compression process (Dhondt et al., 2022a; Hayashi et al., 2021; Paul et al., 2019), the roller compaction process (Yu et al., 2019), etc. At last, the obtained process knowledge or process models can enable in-silico exploring the material properties space and the process parameters space (Dai et al., 2019a), help predict process and product performance, reduce the impact of raw material variability on the drug product quality (Zhang et al., 2019) or avoid potential process failures (Hancock, 2019). As far as we know, the material library approach has not been used in the dry granulation processing route toward tablet preparation.

This paper is a continuation of our previous work (Wang et al., 2022) which focused on the change of tabletability in the wet granulation process. Considering the fact that some materials (e.g. levetiracetam (Kuntz et al., 2011) or C*PharmMannidex 16,700 (Souihi et al., 2013)) exhibited increased tabletability after roll compaction and dry granulation, the phenomenon of increase or loss of tabletability of material after dry granulation were generalized as change of tabletability. The purpose of this article was to use the material library approach to understand the patterns of tabletability change for different materials processed by roll compaction and dry granulation, clarify the potential risk for tablet formulation design via the RCDG manufacturing route. The rest of this paper was organized as follows: Section 2 introduces the details of the material library, the experimental conditions of RCDG and tableting processes, as well as the methods for characterizing tablet quality, compaction behavior and change of tabletability. In Section 3, the physical properties and compaction behavior of 31 materials were studied in detail. Then, the CBCS parameters of granules and ungranulated powders were compared by multivariate analysis. Afterwards, the change of tabletability for different materials were classified. Finally, a risk decision tree and a predictive model were constructed to assess the risk of tablet failure of the materials and aid decision making in RCDG tablet formulation design. Section 4 sums up the paper and gives future research directions.

## 2 Materials and methods

### 2.1 Materials

31 pharmaceutical materials, including 16 pharmaceutical excipients and 15 natural product powders (NPPs), were chosen to build a material library. In order to create a material library with representative samples, commonly used excipients with different functions such as diluents, binders and disintegrants were selected, like MCC, lactose, hydroxypropyl methylcellulose (HPMC), low-substituted hydroxypropyl cellulose (L-HPC), croscarmellose sodium (CCNa), corn starch, D-sorbitol, DCP and sodium bicarbonate (NaHCO_3_). 4 types of MCC and 5 types of lactose were included in the material library to expand the variation coverage of physical properties. 15 batches of NPPs prepared from 12 medicinal plant materials were provided by the Beijing Tcmages Pharmaceutical Co., Ltd. (Beijing, China). All NPPs were manufactured by pretreatment of medicinal herbs, water extraction, filtration, concentration and spray drying and could be used as raw materials for the fabrication of oral solid dosage of Chinese medicine products (Xiong et al., 2022; Yu et al., 2021; Zhang et al., 2022; Zhao et al., 2023). Compared to excipients, NPPs often possessed high hygroscopicity and poor flowability (Li et al., 2018) and could expand the physical property space of the material library. The name, lot number and supplier for each material are provided in Table S1 in Supplementary Materials.

### 2.2 Characterization of powders

Each material used in the material library was subject to a series of characterization techniques to determine their physical properties. Among them, 12 parameters were measured or calculated according to the SeDeM expert system methodology^[39]^, and they were density parameters (bulk density, *D*_b_; tapped density, *D*_t_), compressibility parameters (inter-particle porosity, *Ie*; cohesion index, *Icd*; Carr index, *IC*), flowability parameters (Hausner ratio, *IH*; angle of repose, *AoR*; flow time, *t*”), stability parameters (moisture content, %*HR*; hygroscopicity, %*H*) and uniformity parameters (particle size <50 μm, %*Pf*; homogeneity index, *Iθ*). In addition, other 6 parameters, i.e. the true density (*ρ*_t_), solid fraction for powder (*SF*_p_), particle sizes *D*_10_, *D*_50_, *D*_90_ and *Span* were also used to describe the properties of powders. The testing procedures of above 18 parameters were thoroughly described by Dai et al. (2019b). The characterization data of all materials are from the iTCM database and are shown in Table S2 in Supplementary Materials.

### 2.3 Roll compaction and dry granulation

Roll-compacted ribbons were manufactured using a LGS120 roll compactor (Beijing Longli Tech Co., Ltd., Beijing, China), equipped with a hopper, a horizontal feed screw, two knurled rim rolls (120 mm in diameter and 35 mm in width), and rim roll sealing. During ribbon production, the roll compactor was set to the roll speed of 6 rpm, the feed screw speed of 20 rpm, and the hydraulic pressure of 70 bar, while the roll gap varied according to the feed material properties. In order to avoid sticking, the roll surface was lubricated by magnesium stearate and the temperature of the rolls was controlled by using a circulating water-cooling system. Samples were collected when the material had completely submerged the feed screw and the ribbon production had been stabilized.

The produced ribbons were manually broken into smaller pieces and then fed into a conical mill granulator with a 2.0 mm sieve screen and the impeller speed set at 300 rpm. Granules were collected and were further sieved into fractions of 125–250 μm and 630–850 μm (Grote and Kleinebudde, 2018; Herting and Kleinebudde, 2008; Sun and Himmelspach, 2006) using an automatic shaking screen (ZNS-300, Beijing Xinghelishi Tech & Dev Co., Ltd., Beijing, China). The sieving time was 5 min, and the vibration frequency was 30 Hz. The collected granules were put into a ziplock bag and were stored at room temperature with relative humidity lower than 40% for subsequent investigations.

### 2.4 Compression of tablets

The powdered materials or dry granulated granules were compressed into tablets using a single punch tablet press (C&C600A, Beijing C&C CAMBCAVI Co., Ltd., China) equipped with 10 mm flat faced punches. The applied mean velocity of the upper punch was 28 mm·s^−1^. Tablets of each material were compressed at the weight of 350 ± 5 mg and were manually filled into the die, while powders with bulk densities below 0.35 g·mL^−1^ were compressed with fill weight of 300 ± 5 mg. The compression force was controlled by adjusting the distance between the upper and lower punches and the accurate pressure was measured through a compression force transducer and was displayed on the control panel. Five compression forces in the range of 3 × 10^3^–11 × 10^3^ N (approximately 40–140 MPa) were used to produce tablets with different hardness. The punch surfaces and die walls were lubricated externally with magnesium stearate before each compression. After ejection, tablets were placed in an airtight container for at least 48 h to allow for elastic recovery prior to measurement of dimensions and crushing force.

### 2.5 Characterization of tablets

The tablet thickness (*H*, in mm) and diameter (*D*, in mm) were measured by a digital thickness gauge (547–401 Digimatic Caliper, Mitutoyo, Japan) and the tablet weight (*m*, in *g*) was measured using an electronic balance (GL124-1SCN, Beijing Sanfu Hezhong Technology Development Co., China). The diametrical breaking force (*F*, in N) of a tablet was measured using the tablet hardness tester (YPD-500, Shanghai Huanghai Medicine Inspection Instrument Co., Ltd., China) and the tensile strength (*TS*) of tablet was calculated according to eq. 1 (Fell and Newton, 1970).

(1) $$\mathrm{TS}=\frac{2F}{\mathrm{\pi DH}}$$

where *F*, *D*, *H*, as defined above, were the diametrical breaking force, the tablet diameter and the tablet thickness, respectively.

The solid fraction (*SF*) was calculated based on the tablet weight, dimensions, and the true density of the powder (*ρ*_t_) using eq. 2. *SF* was also called the relative density of the tablets. The tablet porosity (*ε*) could be obtained according to eq. 3.

(2) $$SF=\frac{\rho_{\mathrm{app}}}{\rho_{t}}=\frac{4m}{\pi D^{2}H\rho_{t}}$$

(3) ε=1−SF

where *ρ*_app_ indicated the apparent density of the tablet. *ρ*_t_ was defined as the true density of tablet, which was equal to the true density of the starting material.

### 2.6 Compaction behavior evaluation

Each material was compressed under the conditions described in Section 2.4 and tablet characterizations were carried out according to Section 2.5. The tensile strength and porosity of the tablets under various compression pressures could be obtained. The compression behavior classification system (CBCS) technique was used to evaluated the compression properties of the material (Dai et al., 2019a). In CBCS, the compression and compaction behavior of materials was characterized from three aspects: the compressibility (porosity-compression pressure relationship), the compactibility (porosity-tensile strength relationship) and the tabletability (tensile strength-compression pressure relationship). By fitting equations listed in Table 1, nine CBCS descriptors are calculated for every material. The curve fitting performance was evaluated by the coefficient of determination (R^2^).

**Table 1:** The compression equations used to calculate the CBCS parameters.

Compaction behavior	Equation		Compression parameter	
Compressibility	Heckel	$\ln\frac{1}{\varepsilon }=\mathrm{KP}+A$	*P*_y_,	$P_{y}=-\frac{1}{K}$
	Gurnham	$\varepsilon =\frac{1}{K}\;\ln\;\left(\frac{P}{P_{0}}\right)$	*K*	
	Kawakita	$\frac{P}{C}=\frac{1}{\mathrm{ab}}+\frac{P}{a}$	*a*, *ab*, *b*^−1^	$b^{-1}=\frac{a}{\mathrm{ab}}$
	Shapiro	$\ln\left(\varepsilon \right)=\ln\varepsilon_{0}-\mathrm{kP}-fP^{0.5}$	*f*	
Compactibility	Ryskewitch-Duckworth	$\mathrm{TS}=T_{0}\exp\left(-k_{b}\varepsilon \right)$	*k*_b_	
Tabletability	Power	$\mathrm{TS}=dP^{g}$	*d*, *g*	

### 2.7 Characterization of tabletability change

Currently, there have been four methods for characterizing the change of tabletability: (1) The reworking potential (Malkowska and Khan, 1983), (2) The ratio of slope of the tabletability curve of granules to that of powders (Hein et al., 2008), (3) The ratio of tensile strength of tablets made of granules to that of powders under the same compression pressure (Herting and Kleinebudde, 2007), and (4) the relative change of tabletability (Wang et al., 2022).

**Fig. 1:**
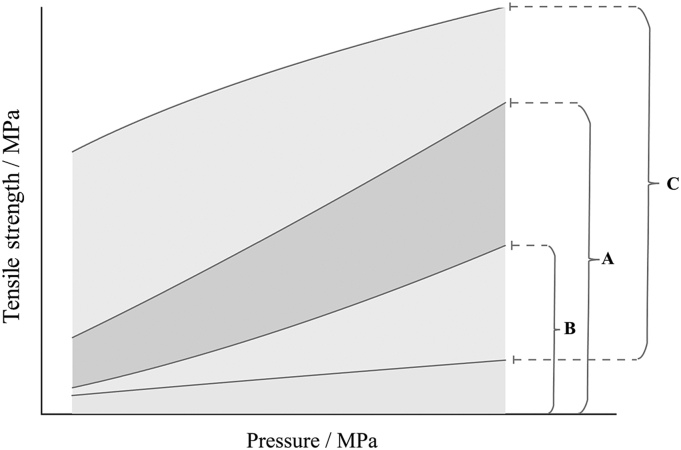
Schematic diagram of the calculation method of reworking potential and relative change of tabletability.

Fig. 1 depicts the calculation method for the reworking potential (*RP*) and the relative change of tabletability (*CoT*_r_). The padding part A is the area under the tensile strength vs. pressure curve of powdered materials, and it is denoted by *AUC*_p_. The padding part B is the area under the tensile strength vs. pressure curve of granules, which is denoted by *AUC*_g_. For a given material, the padding part (B-A) represents its change of tabletability which can be calculated by (*AUC*_g_ - *AUC*_p_).

The area under the lowest tensile strength vs. pressure curve in the material library is defined as *AUC*_min_, and the area under the highest tensile strength vs. pressure curve is defined as *AUC*_max_. The padding part C represents the area between the highest and the lowest tensile strength vs. pressure curves in the material library.

The reworking potential is calculated using eq. 4, which denotes the ratio of area under the tabletability curve of granules to that of powders.

(4) $$\mathrm{RP}=\frac{B}{A}=\frac{{\mathrm{AUC}}_{g}}{{\mathrm{AUC}}_{p}}\times 100\%$$

*CoT*_r_ is calculated by eq. 5, which denotes the ratio of the area between *AUC*_g_ and *AUC*_p_ to the area between *AUC*_max_ and *AUC*_min_.

(5) $${\mathrm{CoT}}_{r}=\frac{B-A}{C}=\frac{{\mathrm{AUC}}_{g}-{\mathrm{AUC}}_{p}}{{\mathrm{AUC}}_{\max}-{\mathrm{AUC}}_{\min}}\times 100\%$$

### 2.8 Multivariate analysis

In this study, the principal component analysis (PCA) was used to reveal latent structures in the data set and identify potential groups of materials. The partial least squares (PLS) regression method was used to develop the relationship between the matrix of independent variables and the matrix of dependent variables. Multivariate data analysis was performed on the SIMCA 14.1 software (Umetrics, MKS, Umea, Sweden).

## 3 Results and discussions

### 3.1 Powder physical properties

**Fig. 2:**
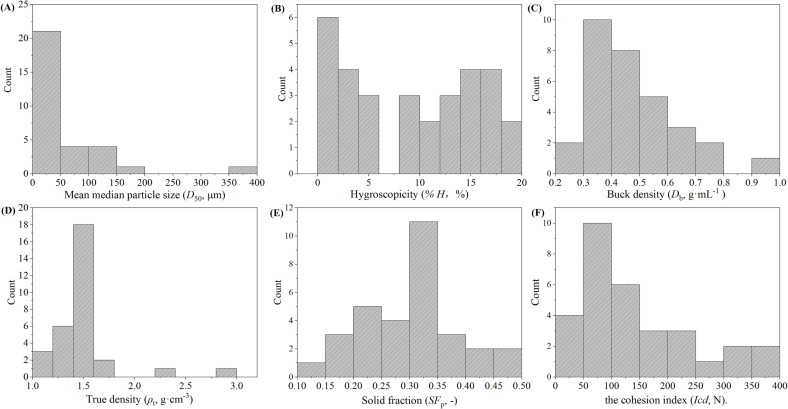
The frequency distribution histograms of six representative powder properties. (A) The mean median particle size (*D*_50_), (B) the hygroscopicity (%*H*), (C) the bulk density (*D*_b_), (D) the true density (*ρ*_t_), (E) the solid fraction for powder (*SF*_p_) and (F) the cohesion index (*Icd*).

As illustrated in Section 2.2, each powdered material was characterized by 18 physical parameters. Six representative parameters were selected as examples to express the diversity of the material library. The histograms of the six parameters are shown in Fig. 2.

The particle size and the *Span* were important factors that affected the compaction behavior of the material (Dai et al., 2019a). It can be seen in Fig. 2(A) that D-sorbitol (No. E15) lying on the far right of the X-axis has the largest median particle size (*D*_50_) of 376.7 μm. The median particle sizes of remaining 30 materials were spread in the range between 8.68 μm (Scutellariae Radix, No. Z15) and 177.2 μm (MCC PH102 SCG, No. E1). Besides, 21 materials in the material library had the median particle sizes <50 μm. Distinct from excipients, which had broad *D*_50_ distribution from 12.6 μm to 367.7 μm, the *D*_50_ values of all NPPs were concentrated in a smaller range between 8.68 μm and 39.6 μm.

As shown in Fig. 2(B), the hygroscopicity values are ranged from near zero (Lactose Flowlac 100 that was abbreviated as Lac F100, No. E5) to 18.97% (Sophorae Flavescentis Radix, No. Z12). Four batches of lactose possessed the hygroscopicity values lower than 1.0%, indicating the excellent stability of lactose materials. The 15 batches of NPPs had relatively high hygroscopicity values that were larger than 9.50%. The high moisture sensitivity of NPPs might be related to the hydrophilic components in the water extracts (Atanasov et al., 2021), as well as their smaller particle sizes and larger specific surface areas.

The bulk density is used to indicate voids within and between particles, and the packing ability of bulk materials. The packing properties of powders varied from the very loosely packed Scutellariae Radix powder (No. Z15, 0.22 g·mL^−1^) to the densely packed DCP powder (No. E16, 0.91 g·mL^−1^). Powders that had higher structural strength and greater inter-particle friction could withstand collapsing, resulting in a lower bulk density. Generally, high bulk density was conducive to improving the powder's manufacturability. For instance, the manufacturing classification system (MCS) summarized that materials with a bulk density >0.50 mL^−1^ were suitable for the direct compression process (Leane et al., 2015).

The true densities of 31 materials were spread in the range between 1.17 g·cm^−3^ (Atractylodis Rhizoma, No. Z8) and 2.91 g·cm^−3^ (DCP, No. E16). Except for DCP, NaHCO_3_ (No. E14) had relatively large true densities that was 2.23 g·cm^−3^. The solid fraction (*SF*_p_) was a measure of solid content of materials and was typically higher for materials with a higher bulk density, but a lower true density. The material with the smallest solid fraction was Scutellariae Radix (No. Z15, 0.15), and the largest was Angelicae Sinensis Radix (No. Z3, 0.47). Powders with larger solid fractions, such as Lac F100 (No. E5, 0.40) and Lactose Anhydrous 21 AN (Lac 21 AN, No. E6, 0.45), were denser and might weaken the plastic deformation ability during compression.

The *Icd* values for all materials varied between 14.3 N (corn starch, No. E11) and 366.8 N (MCC PH102 SCG, No. E1). Within the research scope, six batches of cellulose materials had high *Icd* values that were all >162.9 N. A large *Icd* value suggested a material had adequate compressibility. Yet, the *Icd* values of four kinds of powders were <50 N, and these powders were corn starch (No. E11), Lactose Granulac 200 (Lac G200, No. E7), NaHCO_3_ (No. E14), and DCP (No. E16). Their small *Icd* values might be related to the powder's inability to bond adequately under certain compression conditions (Nofrerias et al., 2018). DCP was demonstrated to be weakly compressible because it was extremely hard to densify below a porosity of 0.3 (Reynolds et al., 2017).

Furthermore, physical properties of 31 materials were combined into a matrix with size [31 × 18]. The dataset was preprocessed using *Z*-score normalization so that different variables were comparable when building models. Then, the PCA was used to perform the dimension reduction and to visualize the high-dimensional data sets. The first three principal components (PCs) successively captured 38.6%, 20.0% and 12.9% of the variability of origin data, respectively. The addition of the fourth principal component did not markedly increase the model's ability to explain the variation, but instead decreased the model's predictive ability (i.e. the Q^2^X value). Therefore, the first three PCs were chosen to construct the PCA model after considering the model's interpretability, predictability, and simplicity.

**Fig. 3:**
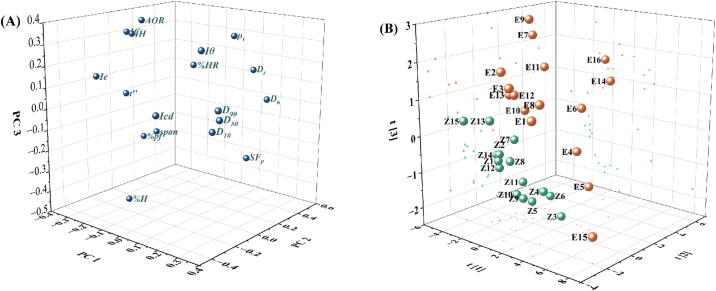
The principal component analysis for the material dataset of 31 materials. (A) The loading plot; (B) the score plot.

The PCA loading plot is a useful tool to show how the original variables contribute to each PC and how different variables are related with each other. As shown in the loading plot (Fig. 3A), the particle size parameters (i.e. *D*_10_, *D*_50_, *D*_90_ and *Iθ*), the flowability parameters (i.e. *AoR*, *t*”, *IH* and *IC*), and the density parameters (i.e. *D*_b_, *D*_t_, *ρ*_t_ and *SF*_p_) are clustered, respectively. The variables in the same cluster are positively correlated. The further away from the plot origin a variable lies, the stronger the impact that the variable has on the model. For instance, the particle size parameters *D*_10_, *D*_50_ and *D*_90_ are scattered on the positive side of the PC1-axis, while the flowability parameters (i.e. *AoR*, *t*”, and *IH*) are scattered on the negative side of the PC1-axis. Since the opposite position of parameters indicated the negative correlation, the materials (e.g., No. E4 or E6) with larger particle sizes may have better flowability. Besides, the density parameters (i.e. *D*_b_ *D*_t_ and *ρ*_t_) and compressibility parameter (*Icd*) contributed to PC2, which was in line with the fact that powders with low density values were easy to be compressed. The flowability and hygroscopicity parameters contributed to PC3 to a large extent. The hygroscopicity parameter was located at a near 90-degree angle with *Icd*, indicating that they were almost uncorrelated. The score plot for the first three PCs is shown in Fig. 3(B). The NPPs in green dots are positioned in the bottom-left part of the latent variable space, while the excipients in yellow dots are widely scattered. The NPPs exhibited clearly different physical properties compared to the commonly used excipients. In consideration of the corresponding relationship between the score plot and the loading plot, the NPPs were observed to preserve small particle sizes, high hygroscopicity and low densities. Overall, the results proved that the material library provided a material physical properties space full of information and diversity.

### 3.2 Compaction behavior of powders

In order to thoroughly characterize the compaction behavior of each powder, different compression equations listed in Table 1 are fitted to derive the CBCS parameters, and the goodness of fit values are provided in Table S3 of the Supplementary Materials.

The relationship between tablet porosity and compression pressure serves as a representation of the compressibility of a powder, which is the capacity to apply pressure to reduce its volume (Joiris et al., 1998). The Kawakita *ab* parameter, also known as the rearrangement index, could be used to indicate the incidence of particle rearrangement at low compression pressures during the initial compression and a high value of *ab* corresponds to a high potential for particle rearrangement (Nordstrom et al., 2009). The *ab* parameters for 31 powdered materials in the material library ranged from 0.06 (Polygoni Multiflori Radix Praeparata, No. Z9) to 0.35 (HPMC). The corn starch had a low *ab* value (i.e. 0.09), which was similar to the *ab* index for the maize starch (i.e. 0.08) reported by Klevan et al. (2010), meaning that the starch material had limited initial particle rearrangement. Besides, most of the NPPs (No. Z1-Z3, Z5-Z14) had *ab* values lower than 0.10, which was inconsistent with findings that a powdered material below the critical threshold of particle size (i.e. 40 μm) was prone to rearrangement (Nordstrom et al., 2009). This phenomenon could be explained by the fact that the rearrangement of NPPs may happen at even lower pressures during the manual filling stage before compression.

The degree of particle fragmentation at low compression pressure (0–50 MPa) was indicated by the index of Shapiro *f*. The values of Shapiro *f* varied substantially from 0.0873 (CCNa) to 0.4202 (Lactose Pharmatose 200 M that was abbreviated as Lac P200M, No. E9). Lac P200M is milled lactose monohydrate with a *D*_50_ value of 46.6 μm, and it has a significant tendency to fragment during compression. The resistance to densification under applied pressure was described by the plasticity parameter *P*_y_, which was calculated from the Heckel equation. The *P*_y_ values for the material library ranged from 74.1 MPa (MCC PH102) to 476 MPa (DCP). Two batches of NPPs (No. Z5, 74.5 MPa; No. Z10, 75.5 MPa) and two batches of excipients (i.e. MCC PH102 and MCC PH302) were classified into soft materials. Most NPPs fell within the category of the moderately hard. The mean *P*_y_ value of NPPs (i.e. 123 MPa) was lower than that of excipients (195 MPa), and the mean *f* value of NPPs (i.e. 0.184) was also lower than that of excipients (i.e. 0.266), indicating that the texture of NPPs was relatively soft and NPPs were not easy to fragment.

The relationship between tablet tensile strength and tablet porosity during the compaction process was described by the R-D equation. The derived *k*_b_ parameter was used to reflect the bonding capacity between particles. A higher *k*_b_ value implies that the powders possess inferior bonding capacity. The MCC PH102 had the lowest *k*_b_ value (4.89), and the NaHCO_3_ had the highest *k*_b_ value (15.6). Generally, the cellulose materials such as MCC, HPMC and L-HPC had favorite compactability properties, since their *k*_b_ values were <9.05. By contrast, the lactose materials and inorganic salts had relatively weak bonding capacities. The *k*_b_ values of NPPs were in the range between 6.76 and 13.2, suggesting that NPPs had slightly weak or moderate bonding capacity within the scope of the material library.

Tabletability is the ability of a material to be densified into a compact with specific strength (Sun and Grant, 2001). Values of the tabletability descriptor *d* from the Power equation varied from 9.26 × 10^−4^ (Mume Fructus, No.Z6) to 1.19 (MCC type 102, No.E2), and values of the pressure sensitivity descriptor *g* varied from 0.249 to 1.68. According to the classification criteria with respect to the parameter *d* (Dai et al., 2019a), the tabletability of different powders can be divided into three categories, as shown in Table 2. 20 materials were distributed in Categories 1 and 2A, suggesting that they had good tabletability. Seven cellulose materials (i.e. 5 types of MCC, HPMC, and L-HPC) could reach tablet tensile strengths higher than 2.0 MPa at compression pressures lower than 50 MPa. The Lac 21 AN (No. E6) belonged to Category 2A material, demonstrating it offered the best tabletability among all grades of lactose applied. The Lac F100 and Lac G200 had Category 2B tabletability. Two types of lactose (i.e. Lac P200M and Lactose Tablettose 80 that was abbreviated as Lac T80), NaHCO_3_ and DCP were classified as Category 2C materials, meaning that they could not meet the requirements for tablet tensile strength (i.e. TS ≥ 2.0 MPa) even at high compression pressures. Particularly, it was observed that the pressure sensitivity descriptor *g* had a certain impact on the classification results. When the *g* value exceeded 1.5, the material could own the Category 2 properties even if the *d* value was <2 × 10^−3^. For example, despite having a *g* value of 1.68 and a *d* value of 9.26 × 10^−4^, Mume Fructus (No. Z6) was classified as a Category 2A material, because it could be applied to produce tablets with TS >2.0 MPa at the compression pressure of 50–100 MPa. This result suggested that the criteria for tabletability classification could be further improved by considering the *g* values of the Power equation.

**Table 2:** The tabletability classification results of 31 materials.

Category	Category criteria	Subcategory	Number of materials
1	*d* ≥ 0.5		6
2	2 × 10^−3^ ≤ *d* < 0.5	2A	14
		2B	6
		2C	4
3	*d* < 2 × 10^−3^		1

### 3.3 Compaction behavior of granules

According to Section 2.3, two fractions of granules were collected for each material. The granules in the size fractions of 125–250 μm and 630–850 μm were called small granules and large granules in the following analysis, respectively. The compression equations in Table 1 were used to derive the CBCS parameters of small granules and large granules, respectively. By comparing the CBCS parameters of the granules with those of ungranulated powders, it was possible to examine how the compaction behavior of the materials changed after RCDG. A further comparison of the CBCS parameters between small granules and large granules could help investigate how granules particle size affected the change of compaction behavior.

#### 3.3.1 From powders to small granules

In order to visualize the compaction behavior changes overall, a combinational dataset [62 × 9] consisted of 9 CBCS parameters for 31 powders and 31 small granules was created. Then, the PCA was performed on this grouped dataset after the data preprocessing. It was found that 43.6% and 34.2% of the variation were explained by the first and second principal components, respectively. The third principal component explained only 7.24% of the variation, and the corresponding eigenvalue was <1. As a result, the first two PCs were chosen for building the PCA model.

**Fig. 4:**
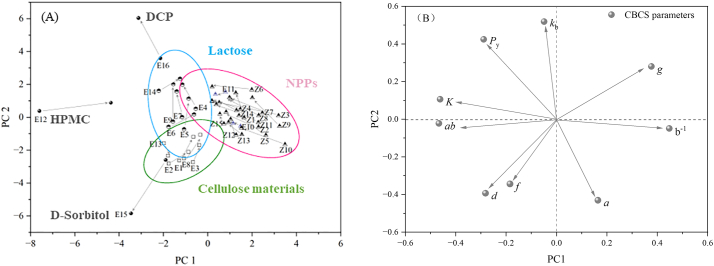
The PCA analysis of the CBCS parameters for raw powders and small granules. (A) The score plot; (B) the loading plot.

Fig. 4(A) shows the score plot of ungranulated powders and small granules, where the arrows represent the compaction behavior change from powders to small granules. As seen from Fig. 4(A), 31 materials are classified into three clusters together with three “outlier” samples. The three clusters were aggregation of cellulose materials, lactose materials and NPPs materials, respectively. The three “outlier” samples were HPMC (No. E12), D-Sorbitol (No. E15) and DCP (No. E16), which had significant changes of compaction behavior in terms of the arrow length. The D-Sorbitol and the L-HPC showed an increase of tabletability since the *d* values of the two materials were increased. The particle sizes of small granules of D-Sorbitol were smaller than the particle size of its ungranulated powders (i.e. *D*_50_ = 376.7 μm). The decreased particle size increased the bonding area of D-Sorbitol granules, resulting in enhanced tablet tensile strength (Castaneda Hernandez et al., 2021). The HPMC possessed the highest *K* and *ab* values, and the arrow pointing at the HPMC granules indicated that the compressibility and rearrangement ability of HPMC were deteriorated after dry granulation. The group of cellulose materials contained 5 materials with good tabletability, plastic deformation ability, and strong interparticle bonding ability. After RCDG, the group of the cellulose materials moved toward the upper right direction in the score plot, losing tabletability and interparticle bonding capacity. However, the locations of powders and granules for cellulose group of materials were closer to the *d* variable than other materials, demonstrating that the LoT did not deteriorate the overall tabletability of them. The brittle materials like DCP and lactose had relatively high *P*_y_ and *k*_b_ values. As revealed by the direction of the arrow, both the compressibility and the bonding capacity of brittle materials were furtherly decreased after dry granulation. Besides, RCDG significantly weakened the plastic deformation and interparticle bonding properties of NaHCO_3_ (No. E14). In the cluster of NPPs were all kinds of NPPs and 2 batches of excipients (i.e. CCNa and corn starch). The NPPs materials were in the positive direction of the PC1-axis, showing that they had good compressibility and plastic deformation ability, but were less likely to be rearranged during the compression process.

#### 3.3.2 From small granules to large granules

Like Section 3.3.1, a combinational dataset [62 × 9] consisted of 9 CBCS parameters for 31 small granules and 31 large granules was created. Then, the PCA was carried out on this dataset after data preprocessing. The PC1, PC2 and PC3 explained 39.9%, 35.5% and 12.5% of the total variance in the data, respectively, and the first two PCs were selected to create a PCA model with 75.4% of the total explained variance.

**Fig. 5:**
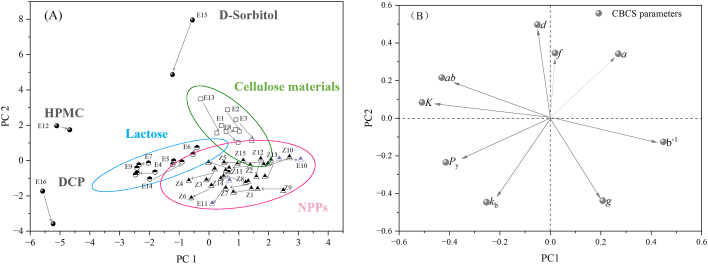
The PCA analysis of the CBCS parameters for small granules and large granules. (A) The score plot; (B) the loading plot.

Fig. 5(A) shows the score plot of the PCA model. Similar to Fig. 5(A), the materials could also be divided into three clusters and three pseudo-outliers. Among the three “outlier” samples, the arrow lengths of D-sorbitol and DCP were very long, revealing a considerable change in their compression behavior. For instance, the bonding force between the large D-sorbitol granules was weakened in comparison to that between the small D-sorbitol granules. And the tabletability of DCP in the form of large granules was better than that in the form of small granules. The compaction behavior of HPMC did not change significantly with increasing granule sizes. For cellulose materials in the green circle, it seemed that the materials were moving toward the lower right side of the latent variable space, and this suggested that the tabletability of cellulose materials decreased and the pressure sensitivity of them increased with increased granule sizes. The lactose materials, however, are not significantly affected by the increase in granule sizes, as evidenced by their short arrow lengths. Particularly, NaHCO_3_ was in the blue circle, and the *P*_y_ and *K*_b_ values of large NaHCO_3_ granules were smaller than those of small NaHCO_3_ granules, implying that the plasticity and bonding capacity were improved after further granule size growth. On the contrary, for NPPs materials in the red circle, the main effects of granule particle size on the materials' compaction behavior were the further reduction in the plasity and bonding capacity.

### 3.4 Classification of tabletability change

In Section 3.3, it was seen that different materials could be clustered according to their compression behaviors. In this Section, the change of tabletability will be thoroughly discussed in terms of classification. Classifying involved using features to sort the material library into different groups or categories, which facilitated us to better understand the relationships between materials.

#### 3.4.1 Qualitative analysis

**Fig. 6:**
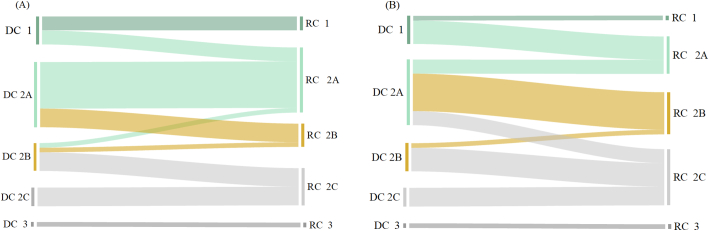
Change of tabletability categories (A) from powders to small granules and (B) from powders to large granules.

The tabletability of both powders and granules could be categorized according to the CBCS classification criteria for the parameter *d*. In cases powders are granulated into small granules, the tabletability categories for 12 materials are altered as shown in Fig. 6(A). The tabletability categories for ungranulated powders and small granules can be seen in Table S4 in Supplementary Materials. Among them, 3 materials, such as MCC PH102 SCG (No. E1), MCC PH102 (No. E3), and MCC PH302 (No. E8), changed the tabletability categories from Category 1 to Category 2A. By contrast, the Scutellariae Radix (No. Z15) displayed an increase of tabletability after RCDG, with its tabletability category being raised from Category 2B to Category 2A. Besides, there were 4 materials whose tabletability categories were changed from Category 2A to Category 2B, and there were 4 materials whose tabletability categories were turned from Category 2B to Category 2C.

In cases powders were granulated into large granules, the tabletability categories for 21 materials were altered after RCDG. The tabletability categories for large granules can be seen in Table S5 in Supplementary Materials. As shown in Fig. 6(B), it is clear that the tabletability categories of more materials (i.e. No. E6, E10, Z1-Z7, Z9, Z12, and Z13) are changed from Category 2A to Category 2B. The tabletability categories for Chuanxiong Rhizoma (No. Z5) and Mume Fructus (No. Z6) were decreased by two levels, i.e., from Category 2A to Category 2C, indicating that the two materials suffered from a high degree of LoT after RCDG. 13 kinds of large granules could not achieve the target of tablet tensile strength (i.e. 2.0 MPa) over the pressure range investigated. The results confirmed that RCDG usually led to tablets with reduced tensile strength, and the risk of tabletability reduction was increased with the granule size enlargement.

#### 3.4.2 Quantitative analysis

**Fig. 7:**
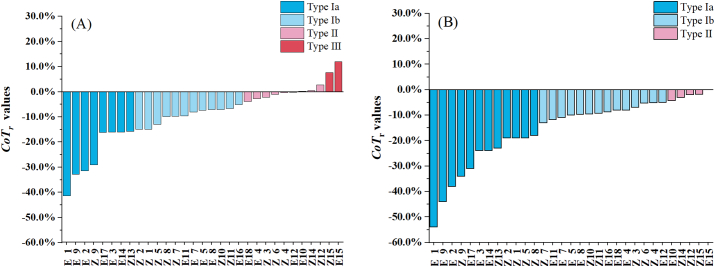
The tabletability change classification system. (A) The *CoT*_r_ values and tabletability change classification results for materials granulated as small granules; (B) the *CoT*_r_ values and tabletability change classification results for materials granulated as large granules.

In order to quantify the degree of change of tabletability, the *CoT*_r_ index was calculated using the methods in Section 2.7. Based on *CoT*_r_ values, the behavior of tabletability change for all materials can be classified into three types, and the proposed tabletability change classification system (TCCS) for RCDG is shown in Fig. 7. The first type (i.e. Type I) was featured by *CoT*_r_ ≤ −5%, and materials falling into Type I had the performance of LoT. The change of tabletability for Type I materials could be further divided into two sub-types, i.e. Type Ia and Type Ib. The *CoT*_r_ values of Type Ia materials were less than or equal to −15%. The materials with *CoT*_*r*_ values ranging from −15% to −5% (including −5%) belonged to Type Ib. In other words, Type Ia materials had larger tabletability reduction than Type Ib materials. The Type II materials was featured by −5% < *CoT*_r_ ≤ 5%. The tabletability of Type II materials had little change after RCDG, and the effect of the change of tabletability on the tablet tensile strength could be ignored. The Type III materials were characterized by *CoT*_r_ > 5%, which meant they had increased tabletability after RCDG.

Fig. 7(A) depicts the *CoT*_r_ values of 31 materials under conditions of being granulated as small granules, which vary from −41.34% to 11.96%. There were 20 Type I materials. Among them, 8 materials were classified into Type Ia, including six batches of excipients and two batches of NPPs. The Type Ia excipients involved 5 kinds of cellulose excipients, such as MCC PH102SG (E1, *CoT*_r_ = −41.34%), MCC type 102 (E2, *CoT*_r_ = −31.40%), MCC PH302 (E8, *CoT*_r_ = −32.78%), MCC PH102 (E3, *CoT*_r_ = −16.06%), and HPMC (E12, *CoT*_r_ = −16.04%). The granule hardening caused by the roll compaction increased the resistance to plastic deformation, thus leading to a large LoT of the MCC materials (Skelbaek-Pedersen et al., 2021). The D-sorbitol (E15, *CoT*_r_ = −16.22%) also belonged to Type Ia. Two batches of NPPs, i.e. Polygoni Multiflori Radix Praeparata (No. Z9) and *Cinnamomum Cassia* (No. Z13), pertained to Type Ia and their *CoT*_r_ values were − 29.31% and − 15.77%, respectively. A total of 12 materials including 5 excipients and 7 NPPs were classified into Type Ib materials. It could be seen that most of lactose (i.e. 3 kinds out of 5), NaHCO_3_ and CCNa belonged to Type Ib powders. The three batches of lactose had similar *CoT*_r_ values, which were − 8.09% (Lac 21AN), −7.33% (Lac F100) and − 7.04% (Lac G200).

9 materials including 4 excipients and 5 NPPs were within the Type II group. The *CoT*_r_ values of Type II materials ranged from −3.85% (DCP) to 2.78% (Sophorae Flavescentis Radix, No. Z12). The excipients belonging to Type II were the remaining 2 batches of lactose (i.e. Lac T80 and Lac P200M), corn starch and DCP. Generally, the tensile strength variation of a Type II material was not prominent before and after the RCDG process. Only 2 materials that were L-HPC (*CoT*_r_ = 11.96%) and Scutellariae Radix (No. Z15, *CoT*_r_ = 7.63%) belonged to Type III. As revealed by the CBCS parameters for the L-HPC, both the compressibility and compactibility of the L-HPC granules were improved by RCDG.

Fig. 7(B) depicts the *CoT*_r_ values of 31 materials under conditions of being granulated as large granules, which vary from −53.94% to −0.06%. 26 materials were classified as Type I, and none of them displayed improved tabletability. 12 materials including seven excipients and five NPPs were furtherly classified into Type Ia. Within this group, five granules such as four batches of MCC (No. E1, E2, E3, and E9) and Polygoni Multiflori Radix Praeparata (No. Z9) had >30% reduction in relative tabletability. Only 5 batches of materials fell into type II. Conventionally, the size enlargement and thereby reduced bonding area between granules were considered as a factor influencing the tabletability of dry granules. In our case, the difference between *CoT*_r_ value for a material granulated as large granules and *CoT*_r_ value for the same material granulated as small granules was calculated, and such difference values for 11 materials including 8 excipients and 3 NPPs were <3%. For instance, the *CoT*_r_ values of Lac T80 granulated as small granules and large granules were − 7.33% and − 8.71%, respectively. This phenomenon indicated that the tabletability of these 11 materials was less affected by a further granule size enlargement during RCDG.

### 3.5 The risk decision tree for material's tabletability in RCDG

**Fig. 8:**
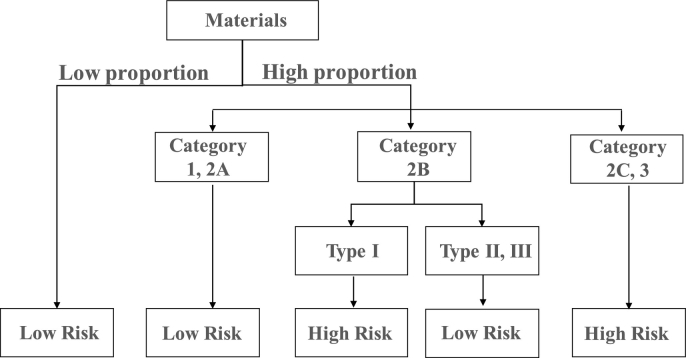
The risk decision tree for evaluation of a material's tabletability in RCDG.

Practically, more than one material will be involved in a tablet formulation, and the effects of additional factors such as the proportion or the function of a component on the final tablet tensile strength should be considered. Based on the comprehensive analysis of the tabletability category and the tabletability change type for all materials in the material library, a decision tree for risk evaluation of a material's tabletability in RCDG is brought forward as shown in Fig. 8. The decision tree is established with small granules data and is validated with large granules data, which are shown in Table S4 and Table S5 in Supplementary Materials.

If the proportion of a powdered material in tablet formulation was low, it may not pose any risk to tablet tensile strength. For instance, the disintegrants such as CCNa and L-HPC in the established material library were typically used in small amounts ranging from 2% to 5% in formulations (Gamble et al., 2010; Jeon and Betz, 2011). Therefore, the contribution of disintegrants to the tabletability reduction of the whole mixture would be weak. Moreover, a study had reported that the intra-granular addition of super disintegrants (i.e. croscarmellose sodium and sodium starch glycolate) could compensate the loss of compactibility of anhydrous lactose-based granules produced via RCDG (Jaspers et al., 2022).

If the proportion of a powdered material such as filler or diluent in tablet formulation was high, the tabletability of powdered materials could help make further decisions when evaluating the potential risk of the occurrence of decreased tabletability. For materials belonging to Category 1 or Category 2A, even if they had a Type Ia or Type Ib change of tabletability, the re-compactibility was still favorable since the produced granules all had good tabletability. In other words, the risk for the occurrence of unwanted tablet tensile strength was low when high proportions of Category 1 or 2A materials were involved in dry granulation formulations. For materials belonging to Category 2C or Category 3, the primary deficiency in tabletability would be inevitably transferred into the intermediate granules, and the high proportion of these materials may result in unacceptable tablet tensile strength.

For materials belonging to Category 2B, it depended on the change of tabletability. If the Type I change of tabletability happened on a material, the risk of tablet failure was high. For example, the tabletability of Notopterygii Rhizoma Et Radix (Z11, *CoT*_r_ = −6.62%) was classified as Category 2B. After RCDG, the tabletability of corresponding granules was classified as Category 2C, which suggested that the tablet tensile strength could not meet the requirements. Nevertheless, If the Type II or Type III change of tabletability happened on a material, there would be no risk to be considered, because the granules still maintained the classification of tabletability of powders after dry granulation.

The tablet categories and tabletability change types for large granules were used to validate the accuracy of the decision tree. For example, the tabletability category of Visci Herba (No. Z2) powder was 2A, and the tabletability change type was Ia. If the Visci Herba was used in high proportion, the related risk was decided to be low. In reality, the tabletability category of Visci Herba granules was 2B, which meant acceptable tablet tensile strength would be obtained at high compression pressure. The judgments were performed for all materials, and the decision accuracy reached 90.3%. For Menthae Haplocalycis Herba (No. Z14), the risk decision result was low, because its tabletability category and the tabletability change type were 2B and II, respectively. Nevertheless, the tabletability category of Menthae Haplocalycis Herba granules was 2C, meaning that acceptable tablet tensile strength (≥ 2.0 MPa) would not be obtained even at high compression pressures. After further looking at the tabletability curve of Menthae Haplocalycis Herba granules, it was found that the tablet tensile strength could reach 1.88 MPa at the compression pressure of 140 MPa. According to the manufacturing classification system (Leane et al., 2015), the tablet with tensile strength ≥1.7 MPa would be robust to further processing. Therefore, the risk decision result for Menthae Haplocalycis Herba material was reasonable. Under the pressure of 140 MPa, two category 2A materials that were wrongly classified as low-risk could obtain tablets with tensile strength >1.7 MPa. Therefore, it could be concluded that the decision tree is reliable in its judgment results. The decision tree for risk analysis of a material's tabletability could be used as a reference for formulation design in the dry granulation and tableting processes. Anyway, for high-risk materials, their proportions in drug product should be paid attention, and a balanced performance was required in the tablet formulation design.

### 3.6 Comparison of the material property design space for DC and RCDG toward tablet manufacturing

With the help of predictive modeling, it was possible to identify the different requirements for material properties in different tablet manufacturing routes, which were DC and RCDG in this paper. Since the granules with size fractions 630–850 μm were not suitable for tablet production, the small granules were used to establish the predictive model. In addition to 18 physical properties and 9 CBCS parameters that described the material properties, *CoT*_r_ and *RP* were also employed as material property descriptors, and the latter two indexes possessed the advantage that they reflected the tabletability change of a material under a given set of process conditions of RCDG. For simulating the tableting process, 5 compression forces (i.e. 3, 5, 7, 9 and 11 kN) within the compression pressure range were defined, and the corresponding table tensile strength could be calculated from the fitted Power equations for either powders or granules. For 31 materials with each material being compressed at 5 different pressures, a total of 155 observations (31 × 5) could be obtained. For each observation, the 29 material properties and the compression pressure are combined as input variables, and the tensile strength of both DC tablet (*TS*_1_) and RCDG tablet (*TS*_2_) are used as output variables, as shown in Table 3.

**Table 3:** The input and output variables for the partial least squares (PLS2) model.

Type of variable		Variables
Input variable	Material properties	*D*_b_*, D*_t_*, ρ*_t_*, Ie, IC, Icd, IH, AoR, t*”*,* %*HR,* %*H,* %*pf, Iθ, D*_10_*, D*_50_*, D*_90_*, Span, SF*_p_
	CBCS descriptors	*P*_y_*, a, b*^−1^*, ab, K, k*_b_*, f, d, g*
	Tabletability change indexes	*CoT*_r_*, RP*
	Compaction pressure	*P*
Output variable	Tablet property	*TS*_1_, *TS*_2_

**Fig. 9:**
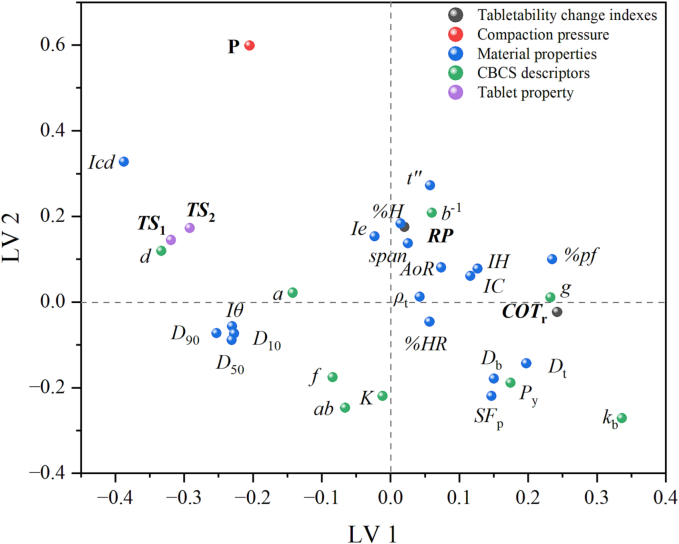
The loading plot for the PLS model based on the first two latent variables.

The PLS2 regression was used to relate the input and output variables. Three indicators, i.e. the fraction of the variation of the X variables explained by the model (R^2^X), the fraction of the variation of the Y variables explained by the model (R^2^Y) and the fraction of the variation of the Y variables predicted by the model (Q^2^Y), were used to evaluate the quality of the model and served as the basis for model optimization. The model metrics for the built PLS model showed that when the first four latent variables were selected, the R^2^Y_cum_ and Q^2^Y_cum_ were 91.9% and 89.5%, respectively, but the R^2^X_cum_ was only 60.9%. The fifth latent variable did not significantly improve the R^2^Y_cum_ and Q^2^Y_cum_ values of the model but could raise the R^2^X_cum_ to 73.5%. Therefore, five latent variables were selected to build the model, and the R^2^Y_cum_ and Q^2^Y_cum_ of the final model were 92.3% and 89.7%, respectively. This demonstrated that the built PLS model had good performance in predicting the tablet quality. Fig. 9 is the loading plot under the first two latent variables. *TS*_1_ and *TS*_2_ were situated near each other, and the correlation coefficient between them was 0.919. This suggested that the compaction of the roller compacted granules to form tablets is closely related to the compaction of the primary particles (Farber et al., 2008; Shi and Hilden, 2017). It can be seen from Fig. 9 that, *Icd*, *d*, and *P* are strongly positively correlated with *TS*_1_ and *TS*_2_. While, the compaction descriptors (*K*_b_, *P*_y_) and the density related parameters (*D*_b_ and *D*_t_) are negatively correlated with *TS*_1_ and *TS*_2_. In addition, the *CoT*_r_ index had a strong negative correlation with *TS*_1_ and *TS*_2_, demonstrating that the *CoT*_r_ index succeeded in predicting the tablet tensile strength. However, the *RP* was located near the center of the loading plot, indicating that it contributed little to the prediction of tablet quality.

**Fig. 10:**
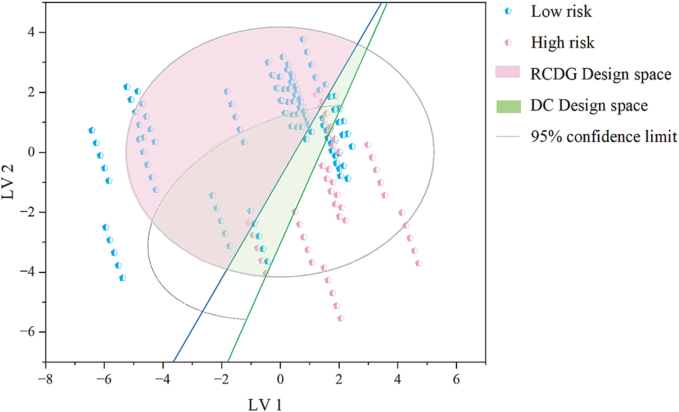
The design space developed based on the PLS model. (The green line represents *TS*_1_ = 2.0 MPa, and the blue line represents *TS*_2_ = 2.0 MPa. The green area represents the DC design space, and the red area represents the RCDG design space.) (For interpretation of the references to colour in this figure legend, the reader is referred to the web version of this article.)

By setting the targets for *TS*_1_ and *TS*_2_ of tablets, the area that met tablet requirements in the latent variable space could be visualized in the score plot. As shown in Fig. 10, the green and blue lines correspond to *TS*_1_ = 2.0 MPa and *TS*_2_ = 2.0 MPa, respectively. The area within the 95% confidence ellipse and above the green and blue lines were regions for DC tablets and RCDG tablets that met the tensile strength requirements, respectively. The semi-ellipse region for DC was larger than that for RCDG, which was attributed to the phenomenon of LoT during RCDG. As discussed in Section 3.1, powdered materials with *D*_b_ higher than 0.5 g·mL^−1^ are suitable for DC. This constraint was added to the score plot by drawing the 95% confidence ellipse on the powders with *D*_b_ higher than 0.5 g·mL^−1^, and the intersectional green area represented the DC design space. This enabled the subdivision of the developability space into areas represented by DC and RCDG. The RCDG design space covered more materials than the DC design space and the RCDG processing route could be used to manufacture tablets with lower *D*_b_ materials. Assuming that all materials were used in high proportions, and the related risk could be estimated by the decision tree in Fig. 8. The materials with a high risk of LoT are represented by red dots in Fig. 10. It could be seen that most of the red dots were distributed on the right side of the target line of RCDG. This demonstrated that the proposed decision tree provided an easy and reliable pathway to a decision, when selecting materials for tablet formulation design. Considering the LoT, selecting the starting materials with good bonding capacity or tabletability was beneficial to achieve the target of tablet tensile strength.

## 4 Conclusion

In this paper, a material library containing 31 materials was built and comprehensively characterized. The univariate distribution analysis and the principal component analysis revealed that the built material database was full of diversity, and the natural product powders complemented the material property space of the pharmaceutical excipients. By applying a moderate roll compression force, the primary powders were designed to be granulated as small granules and large granules, respectively. Both the powders and granules were characterized by 9 CBCS parameters. The changes in the compression behaviors from powders to granules were investigated by the principal component analysis. Generally, the RCDG process deteriorated the tabletability of cellulose materials, the bonding capability of lactose materials and the plastic deformability of natural product materials. A further granules size enlargement did not impact the compaction behavior of lactose materials, but could increase the compressibility and rearrangement ability of natural product materials and decrease the plasticity and bonding capacity of cellulose materials.

The change of tabletability was studied by both the qualitative and quantitative classification methods. With the help of the *CoT*_r_ index, the TCCS was successfully built. The tabletability change of 31 materials could be classified into three groups, i.e. the loss of tabletability, the unchanged of tabletability and the increase of tabletability. Under the conditions of being granulated as small granules, 20 out of 31 materials exhibited the loss of tabletability. While, under the conditions of being granulated as large granules, 26 out of 31 materials showed tabletability reduction. These results demonstrated the prevalent loss of tabletability phenomenon. However, the loss of tabletability of a material may not pose a threat to the final tablet tensile strength. In order to aid decision making in tablet formulation design, a risk decision tree was innovatively developed by the joint application of the CBCS tabletability categories and the TCCS tabletability change types. Attentions should be paid to the Category 2C and Category 3 materials, as well as the Category 2B materials with Type I change of tabletability, when the proportions of these materials were relatively high in tablet formulation. Furthermore, by using data fusion and the partial least squares modeling technique, the material property design space for DC and RCDG could be identified in the latent variable space, which agreed with the risk analysis results.

In this paper, only the single materials and the fixed process conditions were used to explore the tabletability change under RCDG. In practice, both the formulation compositions and the RCDG process parameters could impact the material's change of tabletability. The following studies will investigate the impact of interactions between the material properties space and the process parameters space on the change of tabletability, and construct a more versatile design model for accelerating the tablet formulation and process design via the manufacturing route of dry granulation.

## Funding sources

This research was funded by the 10.13039/501100001809National Natural Science Foundation of China (No. 82074033).

## CRediT authorship contribution statement

**Junhui Su:** Investigation, Formal analysis, Writing – original draft. **Kunfeng Zhang:** Investigation, Validation. **Feiyu Qi:** Visualization. **Junjie Cao:** Data curation. **Yuhua Miao:** Writing – review & editing. **Zhiqiang Zhang:** Resources. **Yanjiang Qiao:** Supervision. **Bing Xu:** Conceptualization, Writing – review & editing, Funding acquisition.

## Declaration of Competing Interest

The authors declare the following financial interests/personal relationships which may be considered as potential competing interests:

Bing Xu reports equipment, drugs, or supplies was provided by We acknowledge the natural product samples provided by Beijing Tcmages Pharmaceutical Co., Ltd.

## Data Availability

I have shared the data at the attach files.

## Appendix A Supplementary data

Supplementary material: Table S1. The information of NPPs and pharmaceutical excipients including names, abbreviations, batch numbers and suppliers. Table S2. Physical property characterization data for all materials in the material library (n=3). Table S3. The compression behavior classification system (CBCS) parameters and the goodness of fit values of corresponding compression equations for each powder. Table S4. Formulation risk analysis for building the risk decision tree. Table S5. Formulation risk analysis for validating the risk decision tree. Image 1
